# Echocardiography-guided percutaneous intramyocardial alginate hydrogel implants for heart failure: canine models with 6-month outcomes

**DOI:** 10.3389/fcvm.2024.1320315

**Published:** 2024-01-15

**Authors:** Hui Ma, Wenqing Gong, D. Scott Lim, Jing Li, Shengjun Ta, Rui Hu, Xiaojuan Li, Minjuan Zheng, Liwen Liu

**Affiliations:** ^1^ Xijing Hypertrophic Cardiomyopathy Center, Department of Ultrasound, Xijing Hospital, Fourth Military Medical University, Xi’an, Shaanxi, China; ^2^ Department of Medicine, Division of Cardiovascular Medicine, University of Virginia, Charlottesville, VA, United States

**Keywords:** percutaneous intramyocardial alginate hydrogels implants, heart failure, animal models, left ventricular function, myocardial contractility

## Abstract

**Background:**

Echocardiography-guided percutaneous intramyocardial alginate-hydrogel implantation (PIMAHI) is a novel treatment approach for heart failure (HF). We validated PIMAHI safety and efficacy in canine HF models.

**Methods:**

Fourteen canines with HF [produced by coronary artery ligation, left ventricular ejection fraction (LVEF) < 35%] were randomised to PIMAHI treatment (*n* = 8) or controls (*n* = 6). Echocardiography, two-dimensional speckle tracking echocardiography, and pathological examinations after a 6-month follow-up were performed. Repeated-measures analysis of variance was used for within-group comparisons.

**Results:**

At 6-month follow-up, PIMAHI treatment reversed LV dilation and remodelling, increasing LV free wall thickness (LVFW, *p *= 0.002) and interventricular septum thickness (IVS, *p *< 0.001) and reducing LV end-diastolic volume (EDV, *p *= 0.008) and end-systolic volume (ESV, *p *= 0.004). PIMAHI significantly improved LV systolic function, increasing LVEF (EF, *p *= 0.004); enhanced LV myocardial contractility, including increased LV global longitudinal strain (GLS, *p *< 0.001), global circumferential strain (GCS, *p *= 0.006), and mitral annulus displacement (MAD, *p *= 0.001). Compared with controls at 6-month, PIMAHI group significantly increased LVFW thickness (8.5 ± 0.3 vs. 6.8 ± 0.2 mm, *p *= 0.002) and IVS (7.9 ± 0.1 vs. 6.1 ± 0.2 mm, *p *< 0.001); decreased LVEDV (30.1 ± 1.6 vs. 38.9 ± 4.5 ml, *p *= 0.049) and ESV (17.3 ± 1.2 vs. 28.7 ± 3.6 ml, *p *= 0.004); increased LV systolic function (42.7 ± 1.5 vs. 26.7 ± 1.1% in EF, *p *= 0.001); and enhanced LV myocardial contractility including GLS (13.5 ± 0.8 vs. 8.4 ± 0.6%, *p *= 0.002), GCS (16.5 ± 1.4 vs. 9.2 ± 0.6%, *p *= 0.001), and MAD (11.4 ± 3.5vs 4.6 ± 2.5 mm, *p *= 0.003). During PIMAHI treatment, no sustained arrhythmia, pericardial, or pleural effusion occurred.

**Conclusions:**

PIMAHI in canine HF models was safe and effective. It reversed LV dilation and improved LV function.

## Graphical Abstract

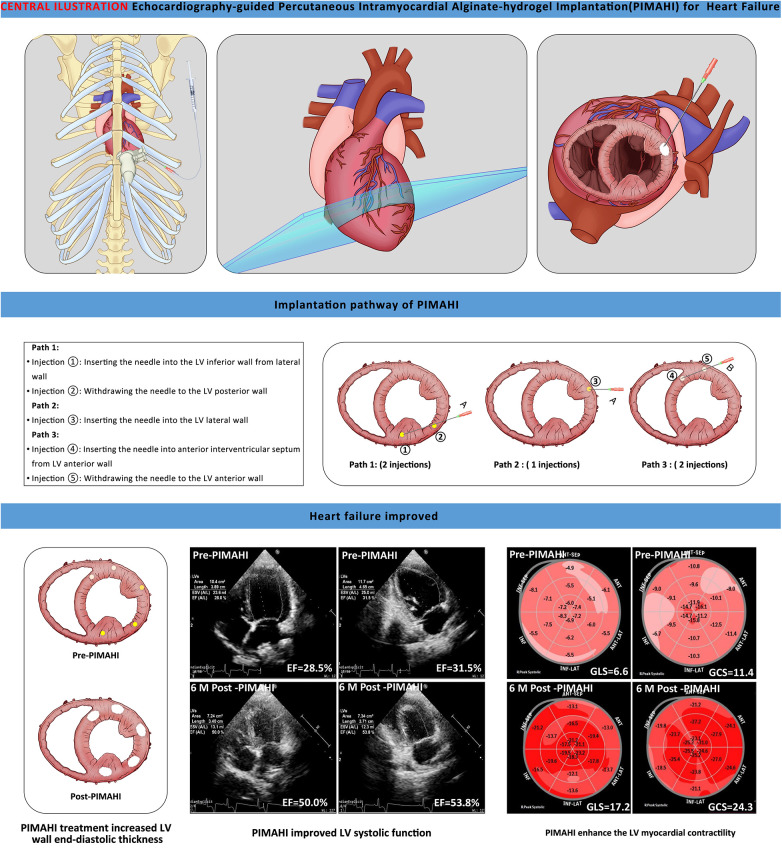

## 1 Introduction

Heart failure (HF) remains a major health problem worldwide and is associated with high mortality and morbidity (1–3). Although patients with HF have improved outcomes with recent advances in drug and device therapy, an estimated 1%–10% of the overall HF population still progress to an advanced stage of the disease, with 5-year mortality rates exceeding 50% (4, 5). As HF progresses, the heart undergoes progressive enlargement of the left ventricle (LV), and its shape transforms from elliptical to one that approximates a sphere with reduced LV systolic function. Improving LV remodelling and LV systolic dysfunction alleviates symptoms and improves the quality of life and prognosis (6, 7).

Alginate is a polysaccharide that occurs naturally in the cell wall of algae and bacterial capsule of Azotobacter sp. and Pseudomonas sp. Because of alginate capability of forming hydrogel, biocompatible, non-toxic materials and non-immunogenic, both alginate and alginate composite have been used for various biomedical applications. Alginate is a natural cytoskeleton with excellent spatial support/filling mechanical properties (8). In several previous studies, direct alginate hydrogel implants (AHI) into the LV wall have been demonstrated to improve the indices of LV systolic function in animal models with advanced HF and to be more effective than standard medical therapy alone for improving exercise capacity and symptoms in patients with advanced chronic HF (9, 10). Previously, AHI was performed with concomitant cardiac surgeries via an open chest procedures. If the hydrogel can be delivered percutaneously, this therapy may become a routine treatment for patients with HF, particularly those who are at high risk for open surgery (11). We developed a novel minimally invasive percutaneous intramyocardial alginate hydrogel implant (PIMAHI) guided by transthoracic echocardiography (TTE) into the LV free-wall, providing a new approach for treating HF with AHI. This study aimed to evaluate the safety and efficacy of a circumferential implant alginate hydrogel using this new minimally invasive approach in canine models of HF.

## 2 Materials and methods

### 2.1 Canine HF models

The local animal ethics committee for research animal care (ethical approval number: 2019120501) approved this study, which was conducted following the ethical standards of the Declaration of Helsinki. The study included 14 canines (male beagles, weight 12.8 ± 0.7 kg, age 1.5 ± 0.2 years old). All canines were obtained from Xi'an Biotechnology (China) and were in good health. The physical examination and echocardiography results of all canines were normal. Fourteen canines underwent surgical ligation of the left anterior descending (LAD) artery below the first and second diagonal branches to produce HF models. They were followed up until the LV ejection fraction (EF) was reduced to 35%. One of the control canines died at 1-month follow-up.

### 2.2 Alginate preparation

Hangzhou Deke MedTech Co., Ltd. (Hangzhou, China) supplied all calcium alginate hydrogel samples prepared sterilely. This hydrogel is considered a “device” used in intramyocardial injections because alginates are inert with no known biological activity in the human body.

### 2.3 PIMAHI procedure

Fourteen canines with HF were randomised to the PIMAHI treatment (*n* = 8) and control (*n* = 6) groups. During PIMAHI, all canines were under general anaesthesia in the right lateral recumbent position, with continuous electrocardiography, blood pressure, and blood oxygen saturation monitoring. The procedure of TTE-guided PIMAHI is shown in the **graphical abstract.** Under the real-time guidance of TTE, a puncture (PTC) needle was inserted percutaneously into the middle segment of the LV wall. Subsequently, along the circumference of the middle LV wall halfway, alginate-hydrogel (Algisyl-LVRTM, supplied by Deke, Inc. Shanghai, China) was injected in five individual points (0.3–0.35 ml per point and the total volume was 1.5–1.75 ml, which achieved similar individual and total hydrogel volume to Sabbah HN.et al' research) (12). The coronary artery branches distributed on the surface of the heart were carefully avoided using colour Doppler ultrasound during implantation.

### 2.4 Echocardiography measurements

Two-dimensional (2D) TTE was acquired at baseline, before PIMAHI, and after PIMAHI (at 1, 3, and 6 months) using an EPIQ 7C/CX50 ultrasound system (Philips Medical Systems, Bothell, Washington) with transducer S5-1 (frequency: 1.6–3.2 MHz) to evaluate the variation in cardiac remodelling and function. The canines were placed in the right lateral recumbent position and underwent TTE studies (including M-mode, 2D, pulse wave, and colour flow Doppler examinations). Electrocardiography (ECG) was simultaneously performed.

Echocardiographic parameters were measured, including end-diastolic wall thickness (EDWT) of the interventricular septum (IVS) and LV free-wall (FW), the amplitude of LV anterior wall motion (AWM) and posterior wall motion (PWM), LV end-systolic volume (ESV) and end-diastolic volume (EDV), E peak of mitral valve flow spectrum/e peak of tissue Doppler mitral valve ring motion (E/e), deceleration time (DT) of early mitral inflow velocity, and length of mitral regurgitation (MR). LVEF and stroke volume (SV) were calculated using the formula recommended by the American Society of Echocardiography guidelines. The end-diastole sphericity index (EDSI) was also calculated (13). LV Global longitudinal strain (GLS) and global circumferential strain (GCS) were analysed using 2D speckle tracking imaging (STI). Mitral annulus displacement (MAD) was determined using 2D tissue tracking (TT). A blinded reader performed all measurements and analyses of echocardiographic parameters.

### 2.5 Gross and pathological examinations

Gross specimens from the PIMAHI and control groups were obtained 6 months after PIMAHI for pathological examination. Transverse slices (parallel to the longitudinal axis of the myocardial fibre) were obtained from the base to the apex level (average 6-mm thickness). Immersion in 10% phosphate-buffered formalin was used to fix them. The fixed tissues were dehydrated, embedded in parafﬁn and sectioned. Histological examination using staining with haematoxylin-eosin (HE) (indicated a myocardial lesion including myocyte hypertrophy, cell arrangement disorder, and interstitial leukocyte inﬁltration) and Masson trichrome (revealed myocardial fibrosis) was performed. Immunohistochemistry was used to detect SERCA1 expression (evaluated myocardial contractility).

### 2.6 Statistical analysis

SPSS 23.0 software was used for analysis. Repeated-measures analysis of variance, with alpha set at 0.05, was used for within-group comparisons. If significance was attained, pair-wise comparisons were made using the t-test between post-PIMAHI and PIMAHI before measurements, with *p* < 0.05 considered significant. Inter-group comparisons were made using a repeated-measures analysis of variance with an alpha set at 0.05 to assess treatment effects. The difference between the PIMAHI and control groups was also evaluated. A t-statistic for two means with significance set at *p *< 0.05 was also used to compare the measures between the base and before PIMAHI. All data are reported as mean ± SEM.

## 3 Results

### 3.1 Baseline and Pre-PIMAHI data of 14 HF canines

Fourteen canine models of HF with LV global remodelling were successfully established 14 weeks after coronary ligation. The baseline characteristics (prior to development of cardiomyopathic state) and pre-PIMAHI measurements of the 14 canine models are summarised in Table 1. Compared to the pre-PIMAHI state, the baseline state of these 14 HF canine models demonstrated a larger LV EDV and ESV (*p *< 0.001), and LV EDWT (*p *< 0.001) and EDSI (*p *= 0.008) were reduced, consistent with LV adverse remodelling. LVEF was decreased (*p *< 0.001), LV wall motion (*p *< 0.001), and MAD were compromised (*p *< 0.001) compared to the baseline state. Strain analysis by STI showed that GLS and GCS were decreased (*p *< 0.001), indicating worsened cardiac function compared to the baseline state.

**Table 1:** Baseline characteristics, prior to ischemic cardiomyopathy and pre-PIMAHI indicators of HF canines (*n* = 14).

Variables	Pre-MI	Cardiomyopathy pre-PIMAHI	*P*-value
HR, bpm	114.6 ± 5.4	119.0 ± 4.0	0.507
Weight, kg	12.8 ± 0.2	14.5 ± 0.5	0.006
Wall thickness (end diastolic), mm			
LV FW	8.3 ± 0.1	7.7 ± 0.3	<0.001
IVS	7.5 ± 0.1	6.8 ± 0.1	<0.001
LV volume, ml			
EDV	22.4 ± 0.9	34.7 ± 2.1	<0.001
ESV	10.4 ± 0.9	23.3 ± 1.4	<0.001
SV	12.0 ± 0.8	11.5 ± 0.7	0.642
LV EDSI	0.3 ± 0.2	0.2 ± 0.0	0.008
Cardiac systolic function (LV)			
EF, %	55.9 ± 1.2	33.0 ± 0.7	<0.001
M-mode, mm			
Anterior	6.4 ± 1.5	1.8 ± 0.3	<0.001
Posterior	9.2 ± 0.9	5.7 ± 0.3	<0.001
GLS, %	16.8 ± 0.8	9.3 ± 0.0	<0.001
GCS,%	21.7 ± 1.1	11.6 ± 0.3	<0.001
MAD, mm	11.9 ± 1.7	5.6 ± 2.1	<0.001
MR(length), cm	0.4 ± 0.6	0.8 ± 0.8	0.147
AV Vmax, cm/s/s	89.0 ± 5.6	76.7 ± 4.2	0.091
Cardiac diastolic function (LV)			
E/e′	9.9 ± 1.3	11.1 ± 1.1	0.484
DT, ms	141.2 ± 12.9	134.2 ± 8.4	0.635

Values are mean ± SEM.

LV, left ventricular; EDV, end-diastolic volume; ESV, end-systolic volume; SV, stroke volume; EDSI, end-diastole sphericity index; EF, ejection fraction; LV, left ventricular; FW, Free wall; IVS, interventricular septum; IVS, interventricular septum; DT, early mitral inflow deceleration time; AV, Aortic Valve; GLS, global longitudinal strain; GCS, global circumferential strain; MAD, Mitral annulus Displacement; MR, mitral valve regurgitation.

### 3.2 Outcome of PIMAHI treatment

**Figure 1:**
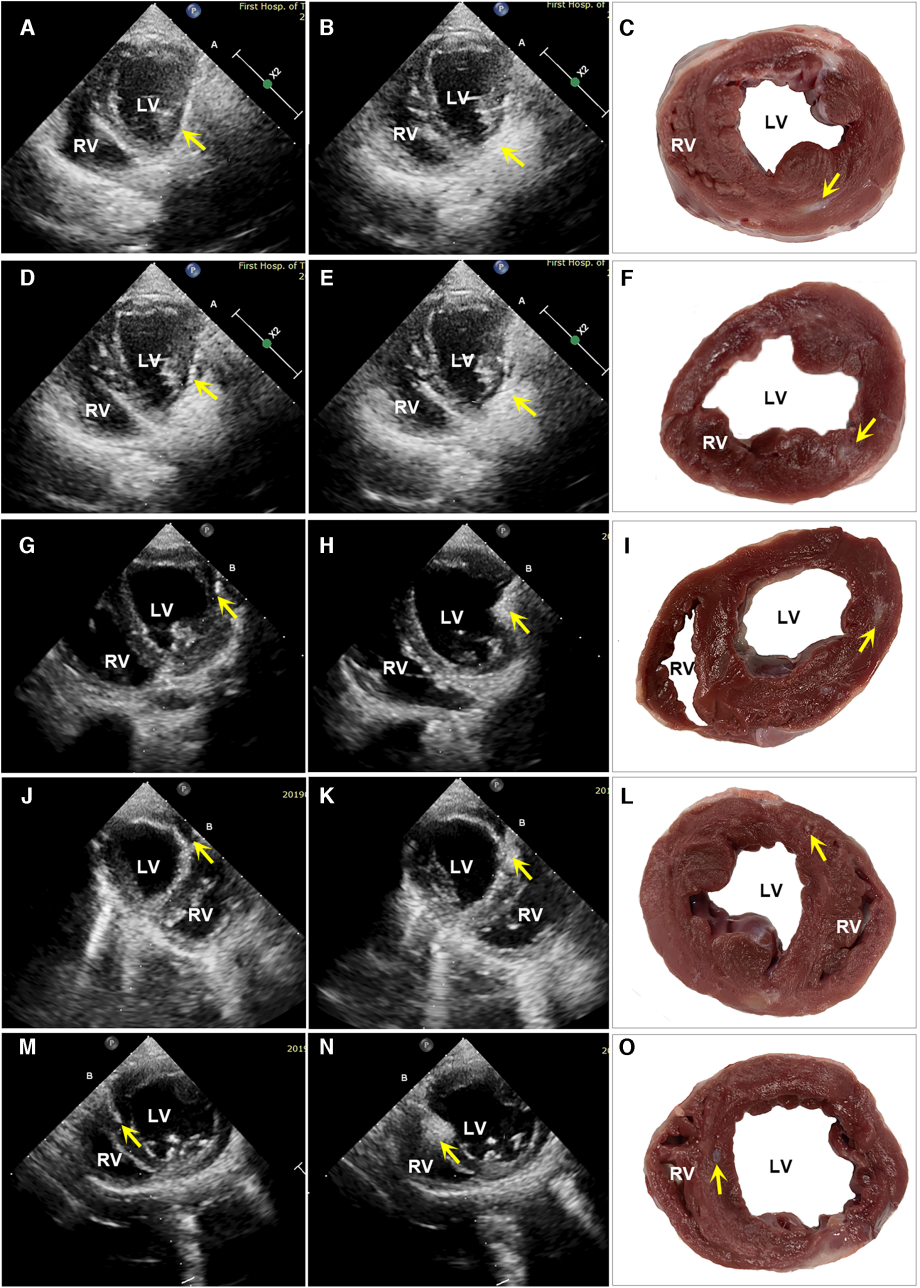
Images of echocardiography and a macroscopic view of heart sections. The left column shows the echocardiographic images of PIMAHI treatment HF canines (**A, D, G, J**, and **M** display the PCT entering IW, PW, IW, AW, and IVSW, respectively, and the PCT tip indicated by arrows). The middle column shows the area echo where the AHI implanted was enhanced (**B, E, H, K**, and **N** displayed IW, PW, IW, AW, and IVSW, respectively, indicated by the arrows). The right column shows the macroscopic view of transverse heart slices of PIMAHI treatment (**C, F, I, L**, and **O**, respectively; AHI pockets in IW, PW, IW, AW, and IVSW, respectively, indicated by the arrows). IW, inferior wall; PW, posterior wall; LW, lateral wall; AW, anterior wall; IVSW, interventricular septum wall. RV, right ventricle; LV, left ventricle.

At 6 months the hydrogel implant were showed remaining within gross specimen of the PIMAHI treatment (Figure 1). During the hydrogel implant procedure, there was no sustained arrhythmia, except for some brief ventricular premature contractions, and no pericardial or pleural effusion was detected. The hemodynamic state of the canine models were maintained, and none required cardioactive medications during the implantation procedure, nor in follow-up.

PIMAHI acutely thickened the LV myocardium at injection sites, reversed LV dilation and remodelling compared to the pre-PIMAHI state—LV EDWT was increased (FW, *p *= 0.002; IVS, *p *< 0.001) and LV cavity size was reduced (LVEDV, *p *= 0.008 and LVESV, *p *= 0.004) in PIMAHI-treated HF canine models (Table 2). In the control group, the follow-up studies showed a decrease in LV EDWT (FW < 0.001; IVS, *p *= 0.002) and an increase in LVEDV (*p *= 0.001) and LVESV (*p *< 0.001), consistent with progression of the cardiomyopathy (Table 2).

**Table 2:** The echocardiographic of LV structure analysis in PIMAHI-treated HF canines and control canines.

	pre-PIMAHI	1 month after PIMAHI	3 months after PIMAHI	6 months after PIMAHI	*P*-value
PIMAHI treated (*n* = −8)					
Wall thickness (end diastolic), mm					
LV FW	7.6 ± 0.1	8.6 ± 0.1*	8.5 ± 0.1*	8.5 ± 0.3*	0.002
IVS	6.8 ± 0.1	7.4 ± 0.1*	7.7 ± 0.2*	7.9 ± 0.1*^,^**	<0.001
LV volume, ml					
EDV	35.9 ± 1.9	30.6 ± 2.0*	30.7 ± 1.8*	30.1 ± 1.6*	0.008
ESV	24.2 ± 1.3	19.2 ± 1.3*	18.8 ± 1.3*	17.3 ± 1.2*	0.004
SV	11.7 ± 0.7	11.4 ± 0.8	11.9 ± 0.8	12.7 ± 0.6	0.078
LVEDSI	0.2 ± 0.0	0.19 ± 0.0	0.2 ± 0.0	0.2 ± 0.0	0.796
Controls (*n* = 5)					
Wall thickness (end diastolic), mm					
LV FW	7.8 ± 0.2	7.4 ± 0.2*	7.1 ± 0.3*	6.8 ± 0.2*^,^**	<0.001
IVS	6.8 ± 0.3	6.6 ± 0.4	6.4 ± 0.6*	6.1 ± 0.2*^,^**	0.002
LV volume, ml					
EDV	32.9 ± 4.7	35.4 ± 3.7	37.4 ± 4.3*	38.9 ± 4.5*	0.001
ESV	21.7 ± 3.1	24.0 ± 2.4*	26.1 ± 3.4*	28.7 ± 3.6*^,^**^,^***	<0.001
SV	11.1 ± 1.7	11.4 ± 1.4	11.3 ± 1.0	10.3 ± 1.1	0.415
LV EDSI	0.2 ± 0.0	0.2 ± 0.0	0.2 ± 0.0	0.2 ± 0.0	0.304

Values are mean ± SEM.

LV, left ventricular; FW, free wall; IVS, interventricular septum; EDV, end-diastolic volume; ESV, end-systolic volume; SV, stroke volume; EDSI, end-diastole sphericity index.

* *p* < 0.05 vs. before PIMAHI.

** *p* < 0.05 vs. 1 month after PIMAHI.

*** *p* < 0.05 vs. 3 months after PIMAHI.

In those canine models who underwent PIMAHI, there was an acute improvement in LV systolic function: LVEF (*p *= 0.004), LV wall motion (AWM *p *< 0.001, PWM *p *= 0.001), and MAD (*p *= 0.001) had increased compared to the pre-PIMAHI state. STI strain analysis showed that LVGLS (*p *< 0.001) and LVGCS (*p *= 0.006) increased significantly in PIMAHI-treated canine models compared with the pre-PIMAHI state. In contrast, LVGLS (*p *= 0.008) and LVGCS (*p *= 0.036) decreased in the control groups (Table 3). Additionally, the cardiomyopathic state in canines resulted in development of MR, which had a significant reduction in the PIMAHI group (*p *= 0.005) (Table 3). In the control group, LVEF (*p *= 0.007) and LV wall motion (AWM, *p *= 0.045; PWM, *p *= 0.013) worsened over time (Table 3). This study demonstrated no significant changes in LV diastolic function in the canine models at any point in time (Table 3).

**Table 3:** The cardiac function analysis in PIMAHI-treated HF canines and control canines.

	pre-PIMAHI	1 month after PIMAHI	3 months after PIMAHI	6 months after PIMAHI	*P*-value
PIMAHI treated (n = −8)					
Cardiac systolic function (LV)					
EF, %	32.4 ± 1.1	37.4 ± 1.4	38.9 ± 1.9*	42.7 ± 1.5*^,^**^,^***	0.004
M-mode, mm					
Anterior	1.5 ± 0.4			3.8 ± 0.4*	<0.001
Posterior	5.4 ± 0.4			8.0 ± 0.3*	0.001
GLS, %	9.5 ± 0.4	14.1 ± 0.5*	13.6 ± 0.6*	13.5 ± 0.8*	<0.001
GCS, %	11.7 ± 0.3	12.3 ± 0.6	14.9 ± 1.3*^,^**	16.5 ± 1.4*^,^**	0.006
MAD, mm	5.0 ± 1.6	8.7 ± 2.3*	9.8 ± 3.0*^,^**	11.4 ± 3.5*^,^**	0.001
MR (length), mm	1.1 ± 0.3	0.2 ± 0.2*	0.3 ± 0.2*	0.2 ± 0.1*	0.005
Cardiac diastolic function (LV)					
E/e′	12.5 ± 1.3			10.9 ± 1.6	0.300
DT,ms	139.1 ± 9.4			117.0 ± 6.3	0.100
Controls (*n* = 5)					
Cardiac systolic function (LV)					
EF, %	33.8 ± 0.8	31.3 ± 0.9*	29.6 ± 0.8*^,^**	26.7 ± 1.1*^,^**	0.007
M-mode, mm					
Anterior	2.4 ± 0.3	-	-	1.3 ± 0.3*	0.045
Posterior	6.2 ± 0.3	-	-	4.1 ± 0.5*	0.013
GLS, %	10.4 ± 0.8	9.8 ± 0.6	9.4 ± 0.8*	8.4 ± 0.6*^,^**	0.008
GCS, %	11.4 ± 0.5	10.0 ± 0.3	9.9 ± 0.4*	9.2 ± 0.6*	0.036
MAD, mm	6.7 ± 2.5	5.9 ± 1.8	5.6 ± 1.8	4.6 ± 2.5	0.542
MR (length), cm	0.3 ± 0.2	1.0 ± 0.2	0.2 ± 0.2	0.4 ± 0.4	0.064
Cardiac diastolic function (LV)					
E/e′	8.7 ± 1.6	-	-	8.6 ± 1.2	0.934
DT, ms	126.2 ± 16.4	-	-	117.4 ± 15.6	0.246

Values are mean ± SEM.

LV, left ventricular; EF, ejection fraction; DT, early mitral inflow deceleration time; AV, Aortic Valve; GLS, global longitudinal strain; GCS, global circumferential strain; MAD, Mitral annulus Displacement; MR, mitral valve regurgitation.

* *p* < 0.05 vs. before PIMAHI.

** *p* < 0.05 vs. 1 month after PIMAHI.

*** *p* < 0.05 vs. 3 months after PIMAHI.

### 3.3 Comparisons of Longer-term PIMAHI treatment effects

**Figure 2:**
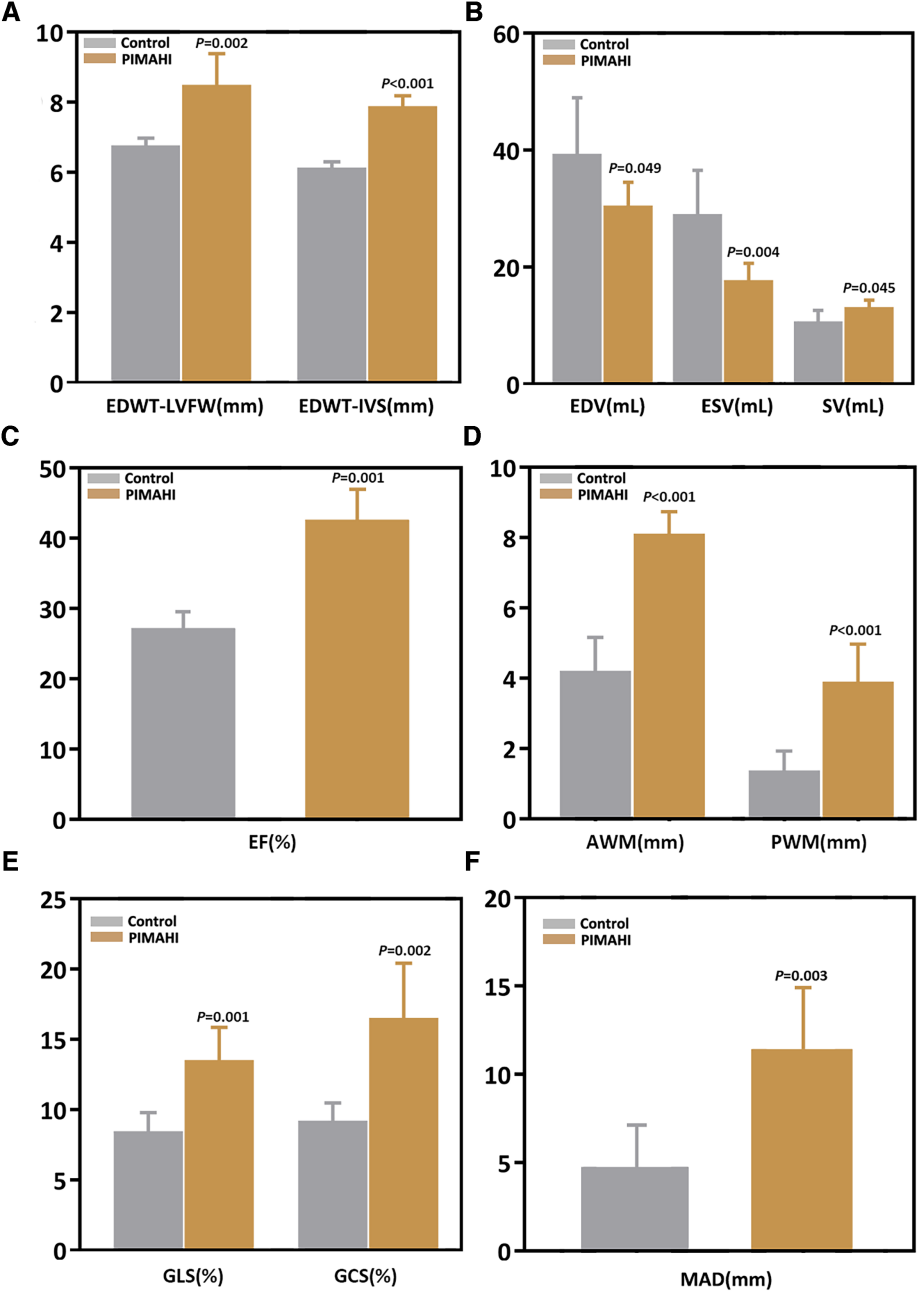
Column chart illustrating the parameter comparison of the two groups at 6 months after PIMAHI. The comparison of EDWT between LVFW and IVS (**A**), LV EDV and ESV (**B**), EF (**C**), AWM and PWM (**D**), GLS and GCS (**E**), and MAD (**F**), in PIMAHI treatment and Control HF canines 6 months after PIMAHI is shown in the column chart. Values are presented as mean ± SEM (*P* < 0.05). PIMAHI, percutaneous intramyocardial alginate hydrogel implant; EDWT, end-diastolic wall thickness; LVFW, left ventricular free wall; IVS, interventricular septum; EDV, end-diastolic volume; ESV, end-systolic volume; SV, stroke volume; EF, ejection fraction; AWM, anterior wall motion; PWM, posterior wall motion; GLS, global longitudinal strain; GCS, global circumferential strain; MAD, mitral annulus displacement.

**Figure 3:**
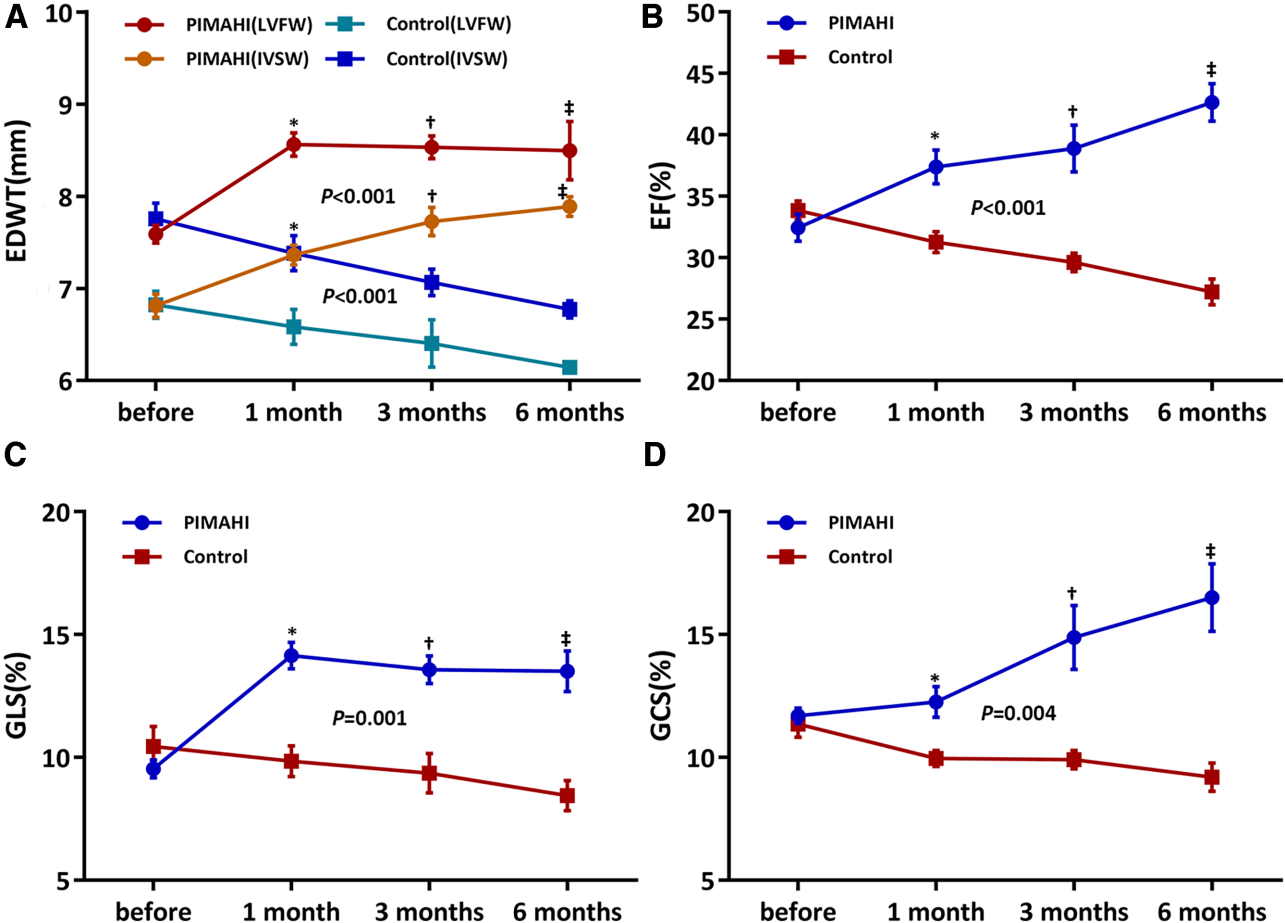
Line graphs illustrating changes in canines. Line graphs illustrate changes in EDWT of LVFW and IVS (**A**), LVEF (**B**), LV GLS (**C**), and LV GCS (**D**) before PIMAHI and 1, 3, and 6 months after PIMAHI compared with the control. Values are presented as mean ± SEM. *P < 0.05, 1 month after PIMAHI vs. the control; ^†^*P* < 0.05, at 3 months PIMAHI vs. the control; ^‡^*P* < 0.05, at 6 months PIMAHI vs. the control. LV, left ventricular; EDWT, end-diastolic wall thickness; LVFW, left ventricular free wall; IVSW, interventricular septum wall; EF, ejection fraction; GLS, global longitudinal strain; GCS, global circumferential strain.

Six months after PIMAHI, the thickness of the LV wall remained increased (Figures 2A, 3A) and LV cavity size had decreased (Figure 2B) of the PIMAHI-treated canine models compared with the controls. When PIMAHI-treated canines were compared to controls at 6 months post-intervention, LVEF and LV wall motion all were increased (Figures 2C–D, 3B). GLS, GCS, and mitral annulus displacement all increased (Figures 2E–F, 3C–D) in PIMAHI-treated canines compared with controls 6 months later.

### 3.4 Pathology and correlation with pathology and correlation with AHI

**Figure 4:**
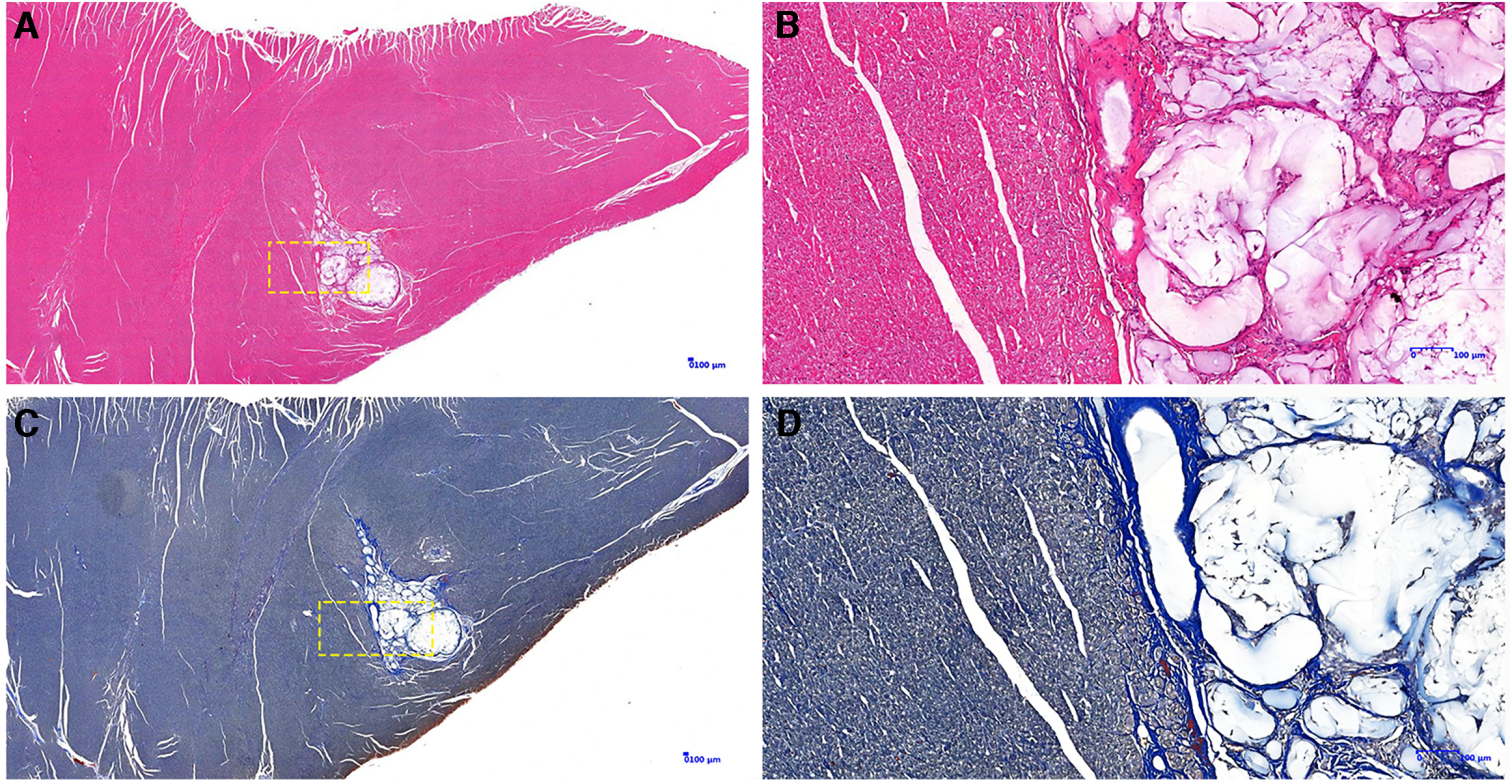
Pathology photographs of sections from the left ventricular free wall in PIMAHI treatment. HE-stained-stained (**A,B**) and Masson-stained (**C,D**) sections showed that the pockets of AHI materials were still within the LV wall, which was encapsulated by a thin layer of connective tissue along with a mild inflammatory response infiltrating lymphocytes and macrophages. (**B,D**) were the yellow dotted box partial zoom image of (**A,C**), respectively.

**Figure 5:**
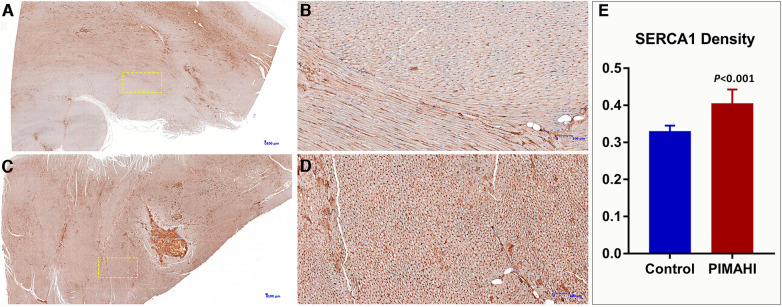
Immunohistochemistry photographs of SERCA 1 expression from the left ventricular free wall in PIMAHI treatment and controls HF canines. SERCA 1 expression increased in the PIMAHI (**C,D**) than in the control group (**A,B**). The sarcoplasmic endoplasmic reticulum Ca2+-ATPase-1 (SERCA-1) density was higher in the PIMAHI group than in the control group (**E**). (**B,D**) were the yellow dotted box partial zoom image of (**A,C**), respectively.

Gross specimens from 8 dogs of the PIMAHI groups, and 5 dogs of the control groups were obtained 6 months after treatment. In PIMAHI-treated cardiomyopathic canine models, HE-stained (Figures 4A,B) and Masson- stained (Figures 4C,D) LV wall sections showed that the pockets of AHI materials were still within the LV wall, which was encapsulated by a thin layer of connective tissue along with a mild inflammatory response infiltrating lymphocytes and macrophages. Adjacent cardiomyocytes showed slight hyaline degeneration. There was no evidence of myocardial toxicity. Immunohistochemistry analysis of PIMAHI treatment showed that sarcoplasmic endoplasmic reticulum Ca2+-ATPase-1 (SERCA-1) expression (Figures 5C,D) increased compared with that in the control group (Figures 5A,B). The SERCA-1 density was higher in the PIMAHI group (*n* = 8) than in the control group (*n* = 5) (Figure 5E), consistent with improvement in myocardial contractility.

## 4 Discussion

HF is a major health problem worldwide and is associated with significant morbidity and mortality, with in advanced heart failure states a more than 50% mortality rate (14). PIMAHI is a new primary interventional approach for treating HF using the AHI. This study demonstrated its safety and efficacy in treating canines with HF, which reduced LV size, partially restored the physiological LV shape, and improved LV systolic function. Intramyocardial AHI therapy as a treatment for advanced HF has resulted in positive initial outcomes in the previous animal and early clinical investigations, including increased LV wall thickness, decreased LV volume and improved LVEF (10–12, 15, 16). In previous studies, all AHI procedures were performed using an open-chest surgical procedure.

PIMAHI is a percutaneous intramyocardial, non-thoracotomy that is accomplished by inserting a PTC needle and implanting the AHI into the myocardium of the LV wall. And this procedure which achieved injection in five individual points along the circumference of the middle of left ventricle wall halfway, has not been reported yet. If confirmed to be feasible and effective, it will provide potential value for the treatment of dilated cardiomyopathy. This minimally invasive approach avoided the physical trauma caused by a sternotomy and the damage to the conduction system distributed underneath the endocardium, and therefore might be safer and more cost-effective. Another advantage of PIMAHI is that the heart beats during the implantation procedure, which reduces ischaemia-reperfusion injury. In addition, PIMAHI percutaneous approach results in an improved cosmetic appearance.

In this study, both during and after the PIMAHI procedure, the circulatory system was not damaged (real-time monitoring of pericardial and pleural effusion by echocardiography and ECG monitoring), as we prevented coronary artery damage by colour Doppler ultrasound before the operation, which might have contributed to the long-term outcomes. Similar percutaneous intramyocardial injection in order to perform radiofrequency ablation in animal studies (healthy sheep models), HCMs, and cardiac tumours by our research team also showed that this procedure is safe (17–20).

There have been studies on the mechanism of action of AHI in treating HF. With some studies demonstrating that, AHI therapy augments the LV wall thickness, due to the filling mechanical properties of alginate, with a subsequent reduction in LV size, which can reduce wall stress based on LaPlace's Law (an indicator of cardiac preload) (11). Other studies have demonstrated that solidified alginate hydrogel material acts as an LV mid-wall constraint skeleton with excellent spatial support. which significantly reduces adverse LV remodelling (14) In this study, the results showed that PIMAHI have thickened the LV myocardium at injection sites, reversed LV dilation and remodelling, consistent with the above mechanism. This present study measured LV strain (GLS and GCS) by STI, which reflects LV myocardial contractility, and mitral annulus displacement by 2D tissue-tracking, which reflects longitudinal contractility of the LV (21–23). The results showed a significant increase of mitral annulus displacement, GLS, and GCS scores at 6 months after PIMAHI treatment. It indicated that AHI in treating HF could improve myocardial contractility. Therefore, this might demonstrate the mechanism of cardiac function improvement and further explain the reason for LV deformation after PIMAHI treatment.

The hydrogen phosphate, dihydrogen phosphate and calcium ions contained in the extracellular matrix of human myocardial tissue are in dynamic balance and the content is very low. Although these ions will undergo ion exchange with the alginate hydrogel implanted in the myocardium, causing the hydrogel to be ion exchanged into a sodium alginate solution, this process is very slow. In addition, the wrapping of fibroblasts will also delay the ion exchange (24). In this study, the pathology examination by microscopy demonstrated that PIMAHI implants remained intact even after 6 months of implantation, which may be relevant to the persistence of the treatment effect. A thin layer of connective tissue encapsulated AHI pockets, which may explain the lack of degradation. There was no evidence of significant inflammation (lymphocytes and macrophages), indicating the safety of AHI treatment. These results are consistent with those of the previous studies. In addition, in this study, immunohistochemistry analysis showed that SERCA 1 expression increased compared with the control group, which reflected the improvement in myocardial contractility and had a favourable impact at the cellular level consistent with reverse LV remodelling (25). SERCA proteins are involved in maintaining calcium homeostasis, abnormalities in the structure or in the amount of which have been mainly related to cardiac malfunction in mammals, so we considered that the improvement of cardiac function may be caused by the increase of calcium uptake capacity which related to SERCA-1 expression increase.

In the initial stage of this study, the needle had occasionally inadvertently entered the heart cavity (6.5%, 3 of 46 points); however, with further experience, we learned to adjust the insertion point to avoid this endocardial puncture. A previous study showed that the intra-vascularly injected hydrogel rapidly dissolved in the bloodstream, and the kidneys excreted the water-soluble alginate chains. There was pathological evidence for adverse effects on the heart, brain, kidney, or liver at 6-month examinations, which further supported the safety of PIMAHI treatment.

## 5 Conclusion and limitations

Our study showed that PIMAHI, guided by echocardiography, as a novel minimally invasive treatment for canines with HF, was safe and effective, as it reversed LV dilation and remodelling and improved LV systolic function.

This was a preliminary study, with a small sample size, and the follow-up period was short. Further enrolment and follow-up of appropriate animals will continue.

## Data Availability

The original contributions presented in the study are included in the article/Supplementary Material, further inquiries can be directed to the corresponding author/s.
