# Supplementary material for: Echocardiography-guided percutaneous intramyocardial alginate hydrogel implants for heart failure: canine models with 6-month outcomes
Source: Front Cardiovasc Med. 2024 Jan 15;11:1320315. doi: 10.3389/fcvm.2024.1320315 (PMC10822984; doi:10.3389/fcvm.2024.1320315)
Supplement: Supplementary file 1 [file Table1.docx]

Supplement Table 1 Comparison of the density of sarcoplasmic endoplasmic reticulum Ca2+-ATPase-1 (SERCA-1) in immunohistochemistry analysis of control and PIMAHI group.

| Group | Number | Area | IOD | AOD | Mean AOD | P value |
| --- | --- | --- | --- | --- | --- | --- |
| Control | Dog 1 | 7262 | 3094.878 | 0.426 | 0.329±0.013 | 0.01 |
|  | Dog 2 | 121618 | 32482.161 | 0.267 |  |  |
|  | Dog 3 | 10187 | 3323.468 | 0.326 |  |  |
|  | Dog 4 | 7265 | 2088.581 | 0.287 |  |  |
|  | Dog 5 | 2821 | 1049.164 | 0.372 |  |  |
| PIMAHI | Dog 7 | 55810 | 22920.36 | 0.411 | 0.404±0.035 |  |
|  | Dog 8 | 102526 | 41303.11 | 0.403 |  |  |
|  | Dog 9 | 49445 | 20134.97 | 0.407 |  |  |
|  | Dog 10 | 66949 | 25572.26 | 0.382 |  |  |
|  | Dog 11 | 92958 | 45149.49 | 0.486 |  |  |
|  | Dog 12 | 160561 | 62854.28 | 0.391 |  |  |
|  | Dog 13 | 64601 | 25353.59 | 0.392 |  |  |
|  | Dog 14 | 76447 | 28060.94 | 0.367 |  |  |

IOD= Integrated option density; AOD=Average optical density
